# Physical Meanings of Fractal Behaviors of Water in Aqueous and Biological Systems with Open-Ended Coaxial Electrodes †

**DOI:** 10.3390/s19112606

**Published:** 2019-06-08

**Authors:** Shin Yagihara, Rio Kita, Naoki Shinyashiki, Hironobu Saito, Yuko Maruyama, Tsubasa Kawaguchi, Kohei Shoji, Tetsuya Saito, Tsuyoshi Aoyama, Ko Shimazaki, Keisuke Matsumoto, Minoru Fukuzaki, Haruchika Masuda, Shinichiro Hiraiwa, Koji Asami, Masayuki Tokita

**Affiliations:** 1 Department of Physics, School of Science, Shonan Campus, Tokai University, Hiratsuka, Kanagawa 259-1292, Japan; rkita@keyaki.cc.u-tokai.ac.jp (R.K.); naoki-ko@keyaki.cc.u-tokai.ac.jp (N.S.); 2 Graduate School of Science and Technology, Shonan Campus, Tokai University, Hiratsuka, Kanagawa 259-1292, Japan; hilonobu.saito@gmail.com (H.S.); greyukoen@gmail.com (Y.M.); tsubasa68k@gmail.com (T.K.); 3 Course of Physics, Graduate School of Science, Shonan Campus, Tokai University, Hiratsuka, Kanagawa 259-1292, Japan; kou3235.msc@gmail.com (K.S.); dunkelhelt0923@gmail.com (T.S.); 3kcat.seal@gmail.com (T.A.); shimazaqui@gmail.com (K.S.); se2gekka@gmail.com (K.M.); 4 Liberal Arts Education Center, Kumamoto Campus, Tokai University, Kumamoto 862-8652, Japan; mfukuzaki@tokai-u.jp; 5 Department of Physiology, Division of Basic Medicine, Tokai University School of Medicine, Isehara Campus, Tokai University, Isehara, Kanagawa 259-1193, Japan; masu3510@tokai-u.jp; 6 Department of Diagnostic Pathology, Tokai University Hachioji Hospital, Hachioji, Tokyo 192-0030, Japan; hiraiwa19@tokai-u.jp; 7 Institute for Chemical Research, Kyoto University, Uji, Kyoto 611-0011, Japan; asami_k2@qc4.so-net.ne.jp; 8 A Graduate School of Science, Kyushu University, 744, Motooka, Nishi-ku, Fukuoka 819-0395, Japan; northbear3.14@gmail.com

**Keywords:** dielectric spectroscopy, aqueous mixtures, open-ended coaxial electrodes, water structures, fractal concept

## Abstract

The dynamics of a hydrogen bonding network (HBN) relating to macroscopic properties of hydrogen bonding liquids were observed as a significant relaxation process by dielectric spectroscopy measurements. In the cases of water and water rich mixtures including biological systems, a GHz frequency relaxation process appearing at around 20 GHz with the relaxation time of 8.2 ps is generally observed at 25 °C. The GHz frequency process can be explained as a rate process of exchanges in hydrogen bond (HB) and the rate becomes higher with increasing HB density. In the present work, this study analyzed the GHz frequency process observed by suitable open-ended coaxial electrodes, and physical meanings of the fractal nature of water structures were clarified in various aqueous systems. Dynamic behaviors of HBN were characterized by a combination of the average relaxation time and the distribution of the relaxation time. This fractal analysis offered an available approach to both solution and dispersion systems with characterization of the aggregation or dispersion state of water molecules. In the case of polymer-water mixtures, the HBN and polymer networks penetrate each other, however, the HBN were segmented and isolated more by dispersed and aggregated particles in the case of dispersion systems. These HBN fragments were characterized by smaller values of the fractal dimension obtained from the fractal analysis. Some examples of actual usages suggest that the fractal analysis is now one of the most effective tools to understand the molecular mechanism of HBN in aqueous complex materials including biological systems.

## 1. Introduction

Recent concepts of dielectric spectroscopy for hydrogen-bonding liquid suggests that the molecular mechanism of a GHz frequency process obtained for aqueous systems is a rate process of cooperative exchange in the hydrogen bond (HB) [1,2,3,4]. Kaatze suggests that the concept in which higher density of the hydrogen bonding network (HBN) brings a decrease in the relaxation time through the increase in the rate constant of the exchange in the HBN should be important, since usages of the HB are often recognized as a factor of restrictions for dynamic molecular behaviors. However, the restriction does not come from the HB itself, and it is brought by decreasing density of the HBN.

**Figure 1:**
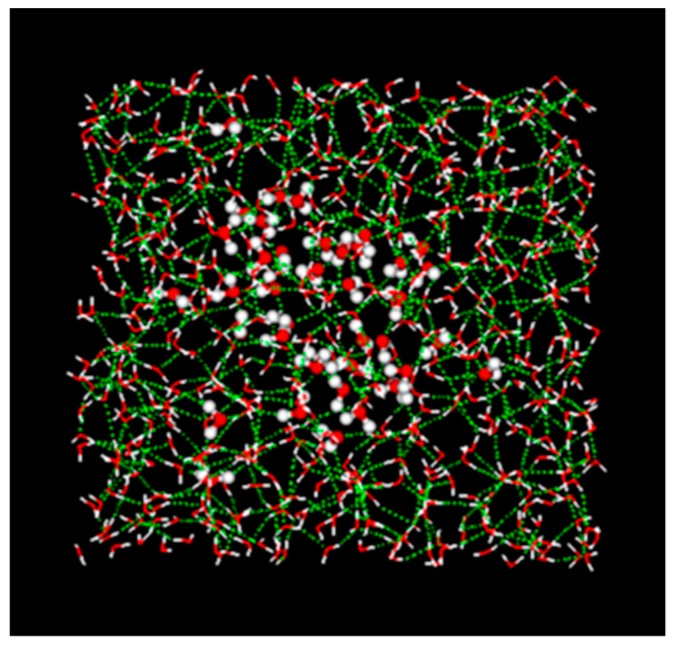
Hydrogen-bonding network (HBN) obtained from a molecular dynamics (MD) simulation. Forty water molecules around the center at the beginning are drawn by van der Walls model and surrounding molecules are shown by the wire model. The MD simulation was performed by Discovery Studio (Biovia) at Tokai University Computing Center.

Figure 1 shows an image of the HBN structure obtained from a molecular dynamics (MD) simulation. It is clearly shown that the HBN is expanded to the whole area. The dynamics of the HBN relating to dynamic liquid structures and behaviors were observed by dielectric spectroscopy measurements as a large relaxation process. The large relaxation process is observed at approximately 20 GHz with the relaxation time of 8.2 ps at 25 °C for pure water [5,6,7]. Aqueous mixtures with rich water contents including biological systems similarly show the process in GHz frequency region [8]. 

**Figure 2:**
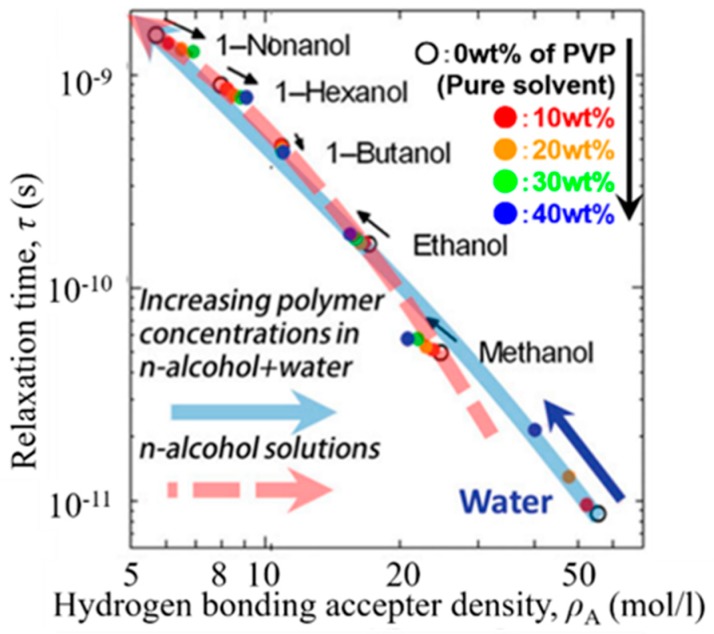
The relaxation time for solvent dynamics versus the HB acceptor density. The open circles indicate values for the pure solvents and closed circles were obtained for 10, 20, 30, 40, and 50 wt% PVP weight fraction. Small arrows indicate the direction of the trajectory with increasing polymer concentration for each solvent. The direction changes between 1-propanol and 1-butanol. Reproduced from Figure 8-4 [3].

It has already been reported that the relaxation time, τ, for various solvents of n-alcohol and water in the solutions of poly (vinylpyrrolidone) (PVP) exhibits systematic changes with the HBN density determined from the PVP concentration and the alkyl chain length of each n-alcohol solvent as shown in Figure 2 [3,4]. Figure 2 indicates that the high HBN density reduces the relaxation time, when the GHz frequency process reflects an increase in the rate of exchanges in higher HBN density. The relaxation mechanism of GHz frequency process is the rate process of cooperative exchanges in the HBN. The HBN density is one of the factors determining the relaxation time, and other series of alcohol molecules with two or three hydroxyl (OH) groups may respectively exhibit different lines.

However, it is not easy to characterize dynamic behaviors especially in the high frequency region because of two major difficulties in observation and analytical techniques in treatments of fluctuations of dynamics at high frequencies. These difficulties have been solved with improvements of measuring techniques with system stability and effective electrodes [9] used at high frequency measurements, and a development of the fractal analysis [10,11,12] has relieved the lack of analytical techniques for a treatment of dynamics fluctuations. The developments in electrodes usually require new designs and those examinations with actual measurements. Furthermore, even in recent investigations with dielectric spectroscopy, however, only average properties of dynamic behaviors of complex materials have been discussed because of a lack of effective treatments of fluctuations and ambiguities in fractal concepts in conventional approaches. Feldman et al. have developed and precisely discussed their models with the fractal concept for more general systems [11,12].

In the present work, a simple and plain characterization of flat-ended coaxial electrodes with the finite element method was examined. The results thus obtained for GHz frequency relaxation processes were analyzed and characterizations of the aggregation and dispersion structures of water molecules were discussed, especially with physical meanings of the fractal natures of the HBN in various aqueous systems. The primitive and simpler model [10] of the fractal concept was treated here, since more extensive application to diversity of aqueous systems was necessary for the present study. The methodologies thus obtained for electrodes design and the fractal concept of water structures are evaluated from phenomenological treatments with physicochemical explanations in the present report. More systematic analyses of detailed designs of electrodes and fractal analyses of water structures with specific properties and functions of respective aqueous complex systems should be treated elsewhere in respective journals in extensive areas.

## 2. Experimental

### 2.1. Materials

Aqueous mixtures examined in the present work were prepared with distilled and deionized water (milli-Q: Millipore Co., Ltd., Tokyo, Japan). Procurements and preparations of various materials have already been reported in our former papers listed in the reference section for each material and the series. Hence, only some details are described here for samples of cell suspensions and organs of mice, for which the authors have not reported sufficiently to date. 

One of authors (K.A.), prepared cell suspensions of yeast strains (Saccharomyces cerevisiae), using yw21-lB (wildtype) and yw21-lA (vacuole-deficient mutant), which were gifted from Y. Ohsumi (University of Tokyo, Tokyo, Japan). Details of sample preparations of the yeast cell suspensions were reported in a former paper [13]. 

Male C57BL/6J mice aged 8-week were purchased from Charles River (Yokohama, Japan) and acclimatized for 2 weeks at a 12 h light-dark cycle. The mice were ad libitum fed with standard laboratory chow (CLEA Rodent Diet CE-2; CLEA Japan, Tokyo, Japan) and conventional water supplied by the animal care facility [14]. 

At the experiment, molecular hydrogen water (H2 water) was adjusted by Aquela hydrogen water 7.0 ppm starter set (Miz Co., Tokyo, Japan). Immediately after transferring H2 water to a glass bottle which can be set to a mouse cage, H2 water was ad libitum fed. Further, H2 water was replaced to the newly adjusted one after 20 and 28 h at the next day. In the meantime after 24 h, lipopolysaccharides (LPS) (Sigma-Aldrich) dissolved in saline (10 mg/kg) was intraperitoneally administered once. 

Twenty hours later after LPS administration, pentobarbital sodium (60–70 mg/kg body weight Somnopentyl; Kyouritu Seiyaku Co., Tokyo, Japan) was intraperitoneally injected to sacrifice for sampling tissues. Degassed water as control water, was adjusted by keeping H2 water in a plastic bottle at room temperature for 24 h.

### 2.2. Methods

#### 2.2.1. Dielectric Relaxation Measurements

Dielectric relaxation measurements were performed by time domain reflectometry (TDR) methods. A Digitizing Oscilloscope Mainframe (HP 54120B) and a Four Channel Test Set (HP 54124A) were employed as the TDR system [15,16,17,18,19,20] to make dielectric measurements especially for a relaxation process due to molecular dynamics of water in the frequency range from 100 MHz up to 65 GHz. 

A 50 Ω semi-rigid coaxial cable (SUHNER) with the outer diameter *d* = 2.2 mmϕ and electric length *γd* = 0.17 mm was typically employed for open-ended electrodes of the TDR measurement. 

#### 2.2.2. Characterization of Open-Ended Coaxial Electrodes

The finite element method (FEM) simulation was performed for the open-ended coaxial electrodes by Multiphysics (COMSOL). Electric field patterns of the fringing field around the surface of the top of open-ended coaxial electrodes were obtained. The field patterns thus obtained were compared with the results of dielectric measurements on a double-layer model with a Teflon block in standard liquids [3]. The coaxial electrodes with outer diameters, 2.2, 3.6 and 6.3 mmϕ, were characterized with a performance probe, 8570E (Agilent Technologies, Tokyo, Japan). Details of the procedure will be reported in our future paper [21].

#### 2.2.3. Fractal Analysis of the Dielectric Relaxation Process 

Generally, dielectric relaxation data are analyzed with relaxation parameters determined by suitable relaxation functions. A typical relaxation function called the Havriliak-Negami equation [22] is extensively applied for various materials including water, liquid molecules, and polymers and is

(1) $$\frac{\varepsilon^{*}-\varepsilon_{\infty }}{\Delta \varepsilon }=\frac{1}{{\left[1+{\left(j\omega \tau \right)}^{\beta }\right]}^{\alpha }}$$

and

(2) 0<α,β ≤1

The complex dielectric constant, ε*, normalized by the high frequency limit of the dielectric constant, $\varepsilon_{\infty }$, and the relaxation strength, Δε, is dependent on the angular frequency, *ω*, with asymmetric and symmetric relaxation time distribution parameters, *α* and *β*, respectively used in Cole-Davidson (*β* = 1) [23] and Cole-Cole (*α* = 1) [24] equations. A single relaxation process without distribution is also expressed by equation 1 with the shape factors, *α* = *β* = 1, called Debye equation [25].

In the case of aqueous solutions of solute molecules with larger molecular weight [24] and hydrogen bonding, small molecules such as methanol and ethanol, often indicate a large symmetric relaxation process with smaller high frequency processes. On the other hand, asymmetric curves are shown for relaxation processes with strongly interacting dipole moments. The physical meanings of these parameters, *α* and *β*, are then respectively referred as chain connectivity of electric dipole moments [26,27] and the density fluctuations with the fractal nature [10,28].

**Figure 3:**
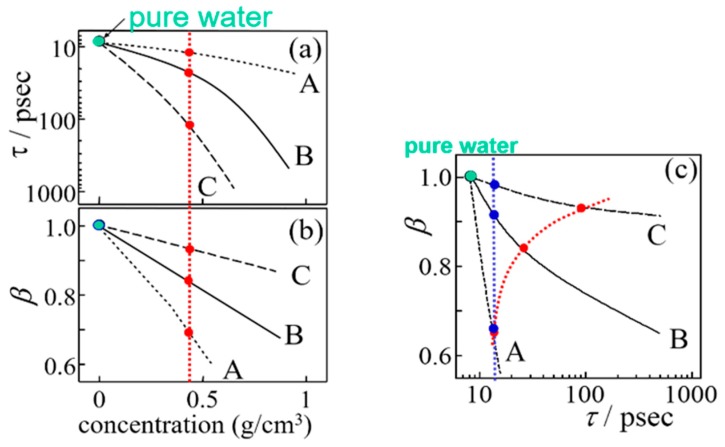
Examples of concentration dependences of (**a**) the relaxation time and (**b**) the relaxation time distribution parameter for model materials, A, B, and C. (**c**) The relaxation time dependent of its distribution parameter for same model materials. Red and blue dotted lines express ideas of the conventional and the present analyses, respectively.

Figure 3 shows our idea of typical materials with different concentration dependences of relaxation parameters for water structures. Typical results are schematically shown as three solid lines for models A, B, and C in Figure 3a,b. Even if the conventional analysis treats the same concentration for comparisons during different materials, it may be difficult to find common properties from each behavior as shown by the red dotted lines in Figure 3 because of the different chemical structures. This study suggests a more universal analysis shown as the blue dotted line in Figure 3c, in which relaxation time distribution parameters are compared at the same value of the mean relaxation time. It means that the relaxation time distribution is analyzed at the same value of normalized mean relaxation time which dynamic behaviors of water molecules are similarly restricted. The *τ* – *β* diagram shows the characteristic behavior of the slow dynamics of water in each material, and some tendencies were obtained for the type of aqueous systems. This universal analysis necessarily required a trajectory of plots of the relaxation time distribution parameters against the mean relaxation time.

Ryabov et al. expressed the relationship between the Cole–Cole parameter, *β*, and the mean relaxation time *τ* in the fractal structure expression [10]:

(3) $$\beta \;=\;\frac{D}{2}\frac{\ln\left(\tau \omega_{S}\right)}{\ln\left(\tau /\tau_{0}\right)},$$

where $\tau_{0}$ is the cutoff time of the scaling in time domain, *D* is the fractal dimension of the point set where relaxing units are interacting with the statistical reservoir:

(4) $$\omega_{S}=2d_{E}G^{2/D}D_{S}/R_{0}^{2},$$

This is the characteristic frequency of the self-diffusion process where *d*_E_ is the Euclidean dimension, *D*_s_ is the self-diffusion coefficient, *R*_0_ is the cutoff size of the scaling in the space, and *G* is a geometrical coefficient approximately equal to unity.

The Equations (3) and (4) have been applied for extensive categories of aqueous systems [3,10]. More developed expressions were successfully applied for HBN structures [11,12]. In the present work, however, the original simple Equation (3) was used for the fractal analysis, since many relaxation processes overlapping to the lower frequency side of the GHz process make it difficult to determine the parameters, such as *ε*_0_ and *ε*_∞_. On the other hand, the relaxation time *τ* and the distribution parameter *β* are obtained without serious difficulties.

## 3. Results and Discussion

### 3.1. Field Patterns of Open-Ended Coaxial Electrodes

**Figure 4:**
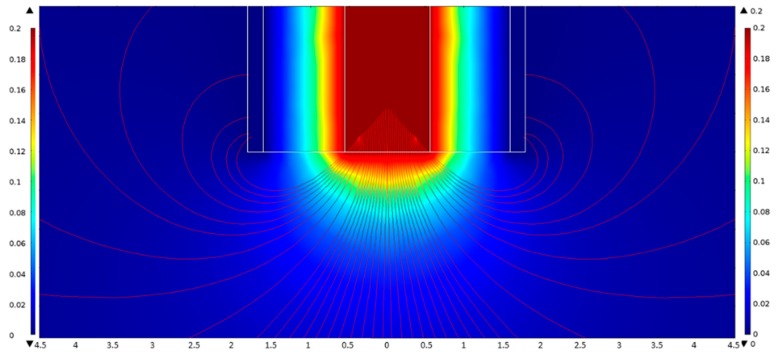
The electric field pattern of the fringing field surrounding open-ended coaxial electrodes with 3.6 mmϕ for the outer conductor. The color indicates the spatial distribution of potential between zero and 200 mV.

Figure 4 shows an electric field pattern of the fringing field around the surface of the top of the open-ended coaxial electrode with 3.6 mmϕ outer diameter of the outer conductor. Values of the high and low potential of 200 mV and zero were set for the inner and outer conductors, respectively. The field pattern was determined by FEM simulation. The spatial distribution of the electric potential was indicated by the gradation of color. 

The field pattern thus obtained was compared with results obtained from dielectric measurements on the double-layer model with a Teflon block in standard liquids [3].

**Figure 5:**
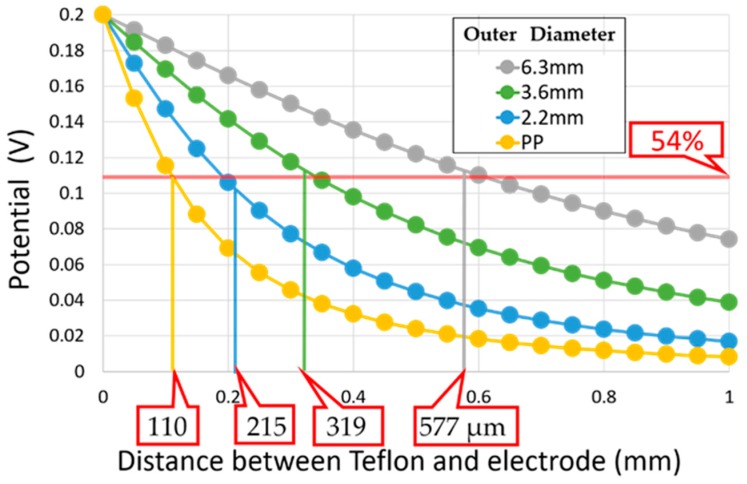
Penetration depth determined from potential obtained by the finite element method (FEM) for each electrode.

The values of electric potential obtained on a vertical line from a center on the top surface of the inner conductor were plotted against the distance from the top surface in Figure 5. The values of the potential showed respective decay curves for coaxial electrodes with various outer diameters. Electrodes with smaller outer diameters exhibit steeper decays as shown in Figure 5.

The field pattern thus obtained was compared with results of dielectric measurements on the double-layer model with a Teflon block in standard liquids [3]. The penetration depth defined by a single exponential function was also indicated in Figure 5 as values shown on the bottom. It is clearly shown that the smaller outer diameter of electrodes with a steeper decay of the potential with increasing distance from the top surface offers a corresponding smaller penetration depth determined from dielectric spectroscopy measurements.

Figure 5 also indicates that the FEM simulation of the potential suggests that the penetration depth can be obtained as the distance from the top surface at which the potential becomes 54% of the highest potential on the surface of the inner conductor. These results suggest a new methodology of a simple and plain procedure of the electrode design by FEM simulations with no actual measurements for characterization of the penetration depth. More detailed procedures and precise results will be reported in future publications [21].

### 3.2. Fractal Analysis with τ − β Diagram

**Figure 6:**
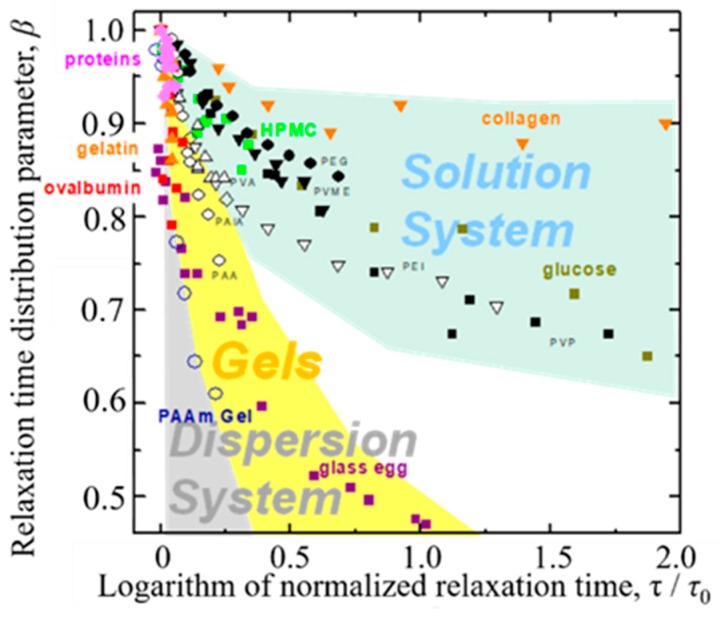
The *τ* – *β* diagram offers fractal dimension, 1 ≤ *d*_G_ ≤ 2 for solution systems and 0 ≤ *d*_G_ ≤ 1 for dispersion systems. Sample names are indicated in the figure with usual abbreviations.

Generally, increasing water content brings about less restricted dynamics of the water molecules in aqueous materials. This property, so called plasticizer effect, is also expressed as slow dynamics for the GHz frequency process of dielectric spectroscopy with an increase in the relaxation time, *τ*, and a decrease in the *β* value. These changes in relaxation parameters also appear as a trajectory in the *τ* – *β* diagram as shown in Figure 6 [3]. Here, the relaxation time was normalized for pure water at 25 °C and simply expressed as *τ*/*τ*_0_. The trajectory of a hyperbolic curve is essentially characterized by three parameters such as two asymptotic lines and a curvature. The horizontal asymptotic line is related to the geometrical fractal dimension and self-similarities of water structures of aqueous materials. The geometrical fractal dimension is induced through the ergodicity in properties of the time response function observed in dielectric spectroscopy [9]. The larger values of *β*-intercept correspond to the higher fractal dimension. However, absolute values of the fractal dimension are not discussed here, since the value is originally determined with the correction parameter [9] and also dependent on the experimental techniques with the characteristic length scales of observation as discussed in the following sections.

On the other hand, all trajectories start from the plot for pure water (*τ* = 8.3 ps, *β* = 1.0 at 25 °C) in the *τ* – *β* diagram (Figure 6), and plots on respective trajectories shift to larger relaxation time and smaller distribution parameter sides with decreasing water content. Water molecules in materials aggregate and form structures with solute molecules, and also dispersed in the mixtures at lower water content. Therefore, trajectories obtained from dielectric spectroscopy make it possible to characterize the slow dynamics of any aqueous systems.

Figure 6 clearly shows that trajectories starting from pure water for aqueous systems with various water contents tend to locate in the upper right area of the *τ* – *β* diagram for aqueous mixtures of polymers [29], such as poly(vinylpyrrolidone) (PVP; weight average molecular weight (Mw) = 10,000), poly(ethylene glycol) (PEG; Mw = 8000), Poly (ethylenimine)(PEI; Mw = 50,000) and poly(acrylic acid) (PAA; Mw = 5000), poly(vinyl methyl ether) (PVME; Mw = 90,000), poly(allylamine) (PAlA; Mw = 10,000), and poly(vinyl alcohol) (PVA; Mw = 77,000). Aqueous solutions of glucose [30] and hydroxypropylmethylcellulose (HPMC) [31] and moist collagen [32] are also shown in this area.

On the other hand, trajectories for protein aqueous solutions of gelatin [33] and ovalbumin [34] with various concentrations and 5 wt% protein aqueous solutions with various molecular weights [35] are located in the lower left area. This tendency of vertical distribution of plots is a characteristic feature of dispersion systems, and our recent studies on cheese and mortar also clearly indicate the same results [36,37] 

In our previous studies, the middle area between those two areas has been offered for gel systems such as poly (acryl amide) (PAAm) aqueous gels [38,39] and glass egg [40], which is obtained from the drying process of thermal-denatured egg white gel. However, the adequacy of the region allocated for gel systems needs to be reconsidered in the present work because of a few examples so far. 

Values of fractal dimension obtained from Equation (3) for the results in Figure 6 suggest that water structures of solution and dispersion systems are respectively expressed by higher and lower fractal dimensions as shown in Table 1. Values of the fractal dimension obtained for 5 wt% protein aqueous solutions show a large variation, since the samples include various kinds of proteins with different molecular weight and even denatured proteins in the gel systems. Despite such large variations, the area of plots is not dispersed much in the diagram, and the variation comes from error in the fitting procedure, because plots of experimental data are essentially only a part of the whole hyperbolic curves. Even in the case of ovalbumin aqueous solutions, the error was still larger than other aqueous solutions. The fractal dimension is corresponding to the *β*- intercept of asymptotic horizontal line, however the value was obtained from fitting procedures of the vertical part of the *τ* − *β* curve, especially for the dispersion systems as shown in Figure 6. Therefore, the error of fractal dimension necessarily tends to be much larger for dispersion systems than solution systems.

Following Figure 6, these results should be interpreted by larger relaxation time distribution parameters, *β*, for solution systems in the upper area, comparing with those for dispersion systems at the same average relaxation time. The low fractal dimension means that water molecules are more heterogeneously dispersed in materials shown in the lower area. Such comparisons of trajectories do not require exact values of the water content and just offer the fractal dimension as 1 ≤ *D* ≤ 2 for solution and 0 ≤ *D* ≤ 1 for dispersion systems, respectively.

### 3.3. Fractal Analysis Dependent on the Scale of Observation

**Figure 7:**
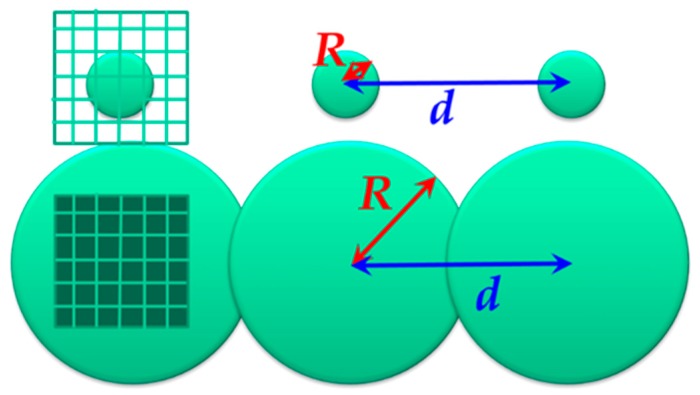
The 100 spheres aligned with the distance, *d* = 1.5. Sphere radii were taken as 0.05 < *R* < 20.

Table 1 also suggests very small fractal dimensions, such as 0.10 for globular proteins. In order to examine if these smaller values are realistic and which physical meanings can be available with the small dimension, box counting methods were applied for geometric models as shown in Figure 7 for 1-dimensional alignment of 100 spheres with radius *R* (0.05 ≤ *R* ≤ 20) and distance, *d* (*d* = 1.5). The geometric model includes various values of fractal dimension for respective length scales. Comparisons of the model with aqueous systems suggest an analogy in which spheres are fragments of the HBN.

**Figure 8:**
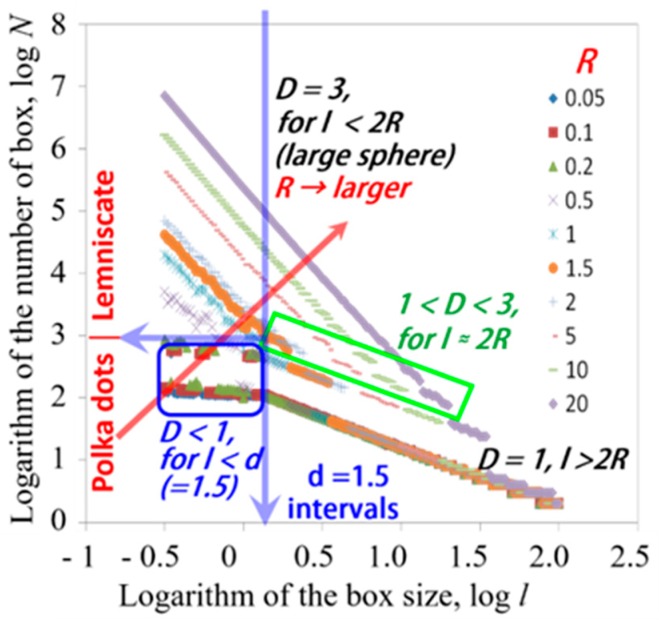
The box size versus number of boxes with spheres. Plots obtained for the radius *R* show straight lines with a change in the slope. The straight lines locate from the below left to the upper right in the figure with increasing *R* from 0.05 to 20.

The results of the box-counting method are shown in Figure 8, in which values of the fractal dimension is expressed as the absolute value of slope of each straight line. Observing those spheres with the smaller box sizes, the fractal dimension must be 3.0 as shown in the upper left region in Figure 8. In the lower right region, the slope changed into 1.0, since the distant vision with the larger box sizes for the sphere alignment is equivalent to a straight line. Furthermore, in the lower left region, very small values of the slope were shown, when the radius *R* is smaller than the intervals and the alignments seem to be dotted lines. Even in this case, the dotted lines seem to be solid lines with the larger box sizes.

This result corresponding to protein solutions means that the HBN dispersed in the solution is fractionated by large protein molecules, and the HBN necessarily shows fragments with very small values of the fractal dimension. Fractal dimensions obtained for collagen, 1.7, was comparable with those for polymer solutions, since the HBN is not excluded by collagen molecules in the fibril, and the HBN takes mutual penetration with collagen molecules, even in the tissues. 

**Figure 9:**
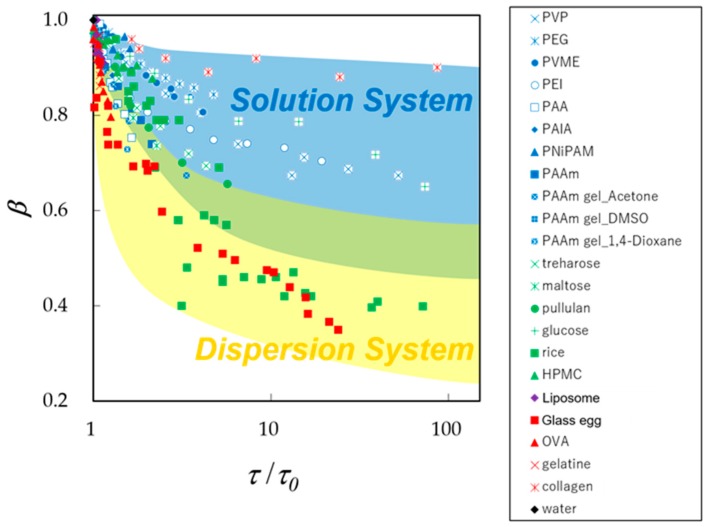
The new *τ* – *β* diagram for various moist materials. Sample names are indicated by abbreviations in the explanatory note.

These results suggest that the fractal dimension is determined with the relation of scales of persistent length of dipole correlation in the HBN and the molecular size of solute and particles in the aqueous systems. If the molecular size of solute in solutions or particle size in dispersion systems are larger than the persistent length of the HBN, then HBN cannot keep network structures and is fractionated as shown in protein solutions. The fractal dimension of the HBN does not take any characteristic values and area for gel systems. The *τ* – *β* diagram thus reconsidered by the fractal concept should be drawn as Figure 9. Plots and trajectories for solution and dispersion systems may overlap in the middle area.

Figure 9 shows more trajectories of various aqueous mixtures. the trajectories for various saccharides [30], such as trehalose, maltose, and pullulan [41], appear in lower region of upper area with increasing molecular weight. This result is considered to be reasonable, since saccharides increase the network size of the HBN with increasing molecular weight. The trajectories for l,2-dipalmitoyl-sn-glycero-3-phosphocholine (DPPC) liposome [9,42] are dense in the left region like those obtained for proteins. the trajectory for rice [43] seems to be exchanged from the upper area for water rich samples to the lower area for water poor samples. This result actually reflects preparation procedures for rice samples with various water contents, as rice kernels absorb a little amount of water, and kernels need to be ground or boiled for water rich samples, therefore native water structures of dispersion systems can be changed. 

PAAm gels with aqueous mixtures of organic solvents, acetone, dimethyl sulfoxide (DMSO), and 1,4-dioxane are also included [44] in Figure 9. The trajectories for these gels also offer detailed physical meanings of the *τ* – *β* diagram with data manipulations of the relaxation time for mix solvents as described in the following section.

### 3.4. Application of the Fractal Analysis for Solvent Molecules in Gel Materials

**Figure 10:**
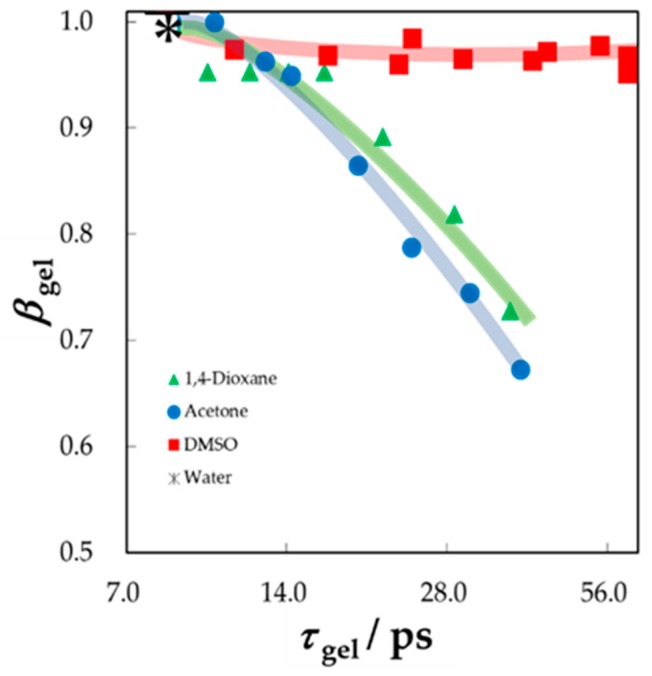
The *τ* – *β* diagram for solvent mixtures in gels. The symmetric relaxation time distribution parameter *β*_gol_ of Cole-Cole equation was used for aqueous solutions of 1, 4-dioxane and acetone, and the Havriliak-Negami equation was used for aqueous solutions of dimethyl sulfoxide (DMSO).

Recently, the authors reported how dynamic behaviors of solvent molecules in gels reflect the volume change [44]. Figure 10 shows the *τ* – *β* diagram for aqueous mixtures of various organic solvents in those gels of PAAm. The volume of gel is generally determined by the solvent [45,46], and PAAm gels often show large volume changes with solvent mixtures. The symmetric relaxation time distribution parameter, *β*_gel,_ was obtained from the Cole-Cole function for aqueous solutions of acetone and 1,4-dioxane in gels. On the other hand, the *β*_gel_ values were obtained from the symmetric distribution parameter of the Havriliak-Negami function for aqueous solutions of DMSO in gels. The composition dependence of relaxation parameters suggest how dynamics of solvent molecules are restricted in polymer networks [45,46,47,48,49,50]. The trajectories reflect, not only dielectric behaviors of solvent mixtures themselves, but also restriction in shrinking gels with decreasing water content. However, trajectories of plots shown in Figure 10 exhibit complicated curves, for which the dispersion of the plots were not simple errors.

**Figure 11:**
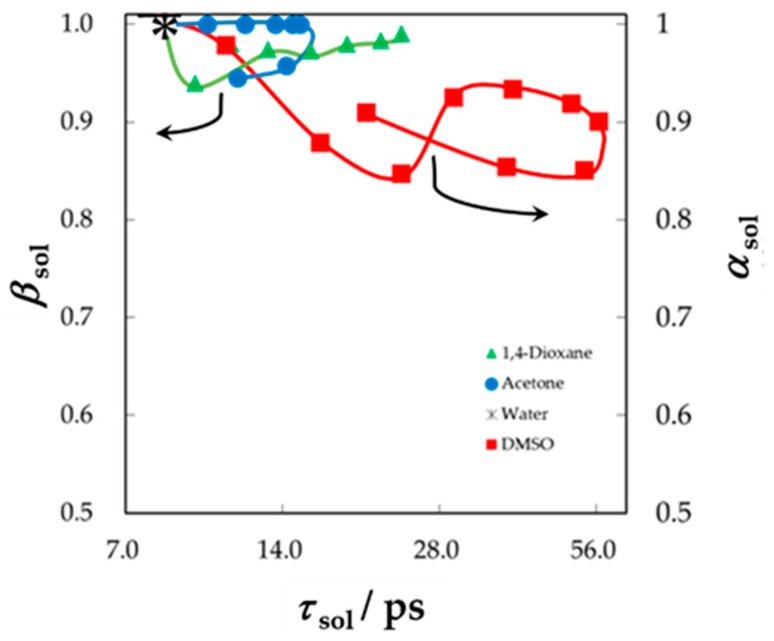
The *τ* – *β* diagram for bulk solvent mixtures. The relaxation time distribution parameter *β*_sol_ of the Cole-Cole equation was used for aqueous solutions of 1,4-dioxane and acetone, and the parameter *α*_sol_ of the Cole-Davidson equation was used for aqueous solutions of DMSO.

Figure 11 shows the *τ* – *β* diagram obtained only for bulk solvent mixtures and clearly indicates trajectories with reentrant behaviors of relaxation parameters with the composition changes. The complicated curves shown in Figure 10 originated from these composition dependences of the bulk solvent mixtures. A GHz frequency process was observed in these binary liquids. The Cole-Cole function was used to represent symmetric relaxation curves for aqueous solutions of acetone and 1,4-dioxane in the whole concentration range, and the Cole-Davidson equation was suitably used to represent the asymmetric curves for aqueous solution of DMSO. Even in the case of DMSO-water mixtures, the asymmetric shape parameter, *a*, was used for the *τ* – *β* diagram in Figure 11.

**Figure 12:**
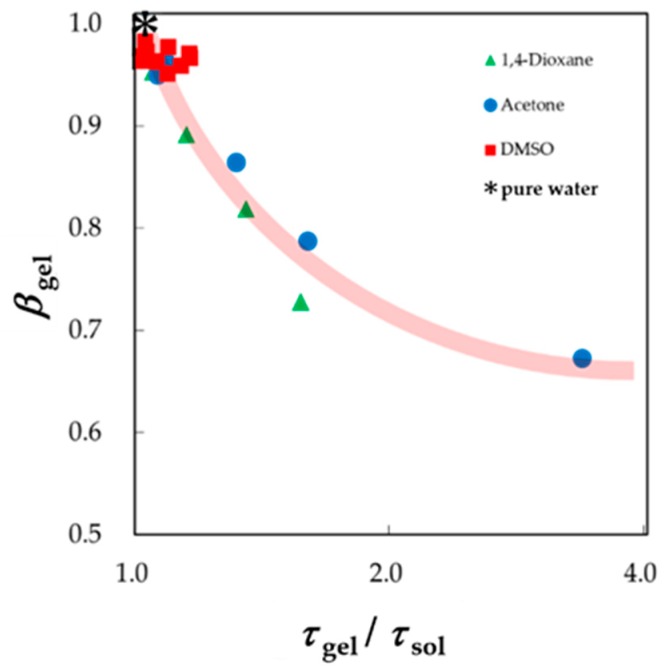
A new *τ* – *β* diagram for solvent mixtures in gels. The relaxation time is normalized, similar to that obtained for bulk solvents.

This study tentatively normalized the relaxation time for solvent molecules in gels by that obtained for bulk solvents. The usual shapes of the *τ* – *β* diagram are obtained as shown in Figure 12. This normalization procedure removes the properties of solvent mixtures, and only the effect of restriction in shrinking gels can be extracted. The trajectories thus obtained mean only the effect of restriction from the volume change, and the restriction is equivalent to the restriction by changing the water content shown by trajectories in the *τ* – *β* diagram. Therefore, trajectories thus obtained exhibit similar curves with usual solution systems as shown in Figure 9. Trajectories thus obtained are located in the area for solution systems, since the HBN and the polymer network are mutually penetrating on similar length scales in the case of the PAAm gels. 

### 3.5. Application of the Fractal Analysis as a Simple Image Analysis

**Figure 13:**
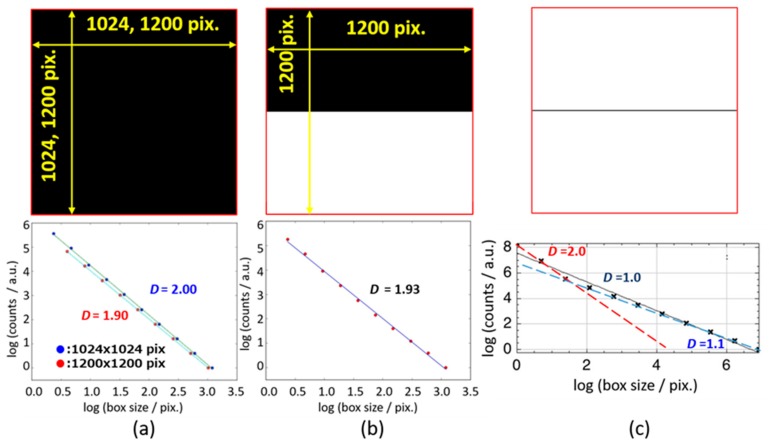
The fractal dimension obtained by box counting method for model systems; (**a**) 2-dimensional full-filled box, (**b**) half full-filled box, (**c**) solid straight line.

However, it is difficult to recognize the fractal dimension directly. Figure 13 shows the geometric models which show how the fractal dimension reflects aggregation and dispersion structures. The value of the fractal dimension obtained for a 2-dimensional box is dependent on the pixel number of how boxes are divided during calculations of box counting. The results indicated in Figure 13 suggest that the D values can be obtained within the error of ± 0.1.

Figure 13a,b suggests that the result obtained for a half full-filled box indicates similar value of the fractal dimension with a full filled box, since the half area brings only a shift of the straight line in the logarithmic scale of box counting procedures and the value of slope is not affected. Furthermore, Figure 13c indicated two different slopes of the logarithmic plot for the fractal dimension, 1.1 and 2.0 for a model line. Here, the D value of 2.0 was obtained, when the scale of observation becomes smaller than the line width of 2 pix. This result will again clarify the scale dependency of the fractal dimension, and the sample is characterized by the size at a discontinuous point of slopes. Especially in the case of experimental techniques to determine the fractal dimension, the discontinuous point of the slopes should be closely related with the scale of observation as pointed out in Figure 8.

### 3.6. Application of the Fractal Analysis for Biological Materials

Biological materials are typical examples of complex materials including water molecules, and the fractal analysis should be effectively applied. As water is the most ubiquitous material, a universal analysis of the HBN must be suitable for an extensive area of usage. 

**Figure 14:**
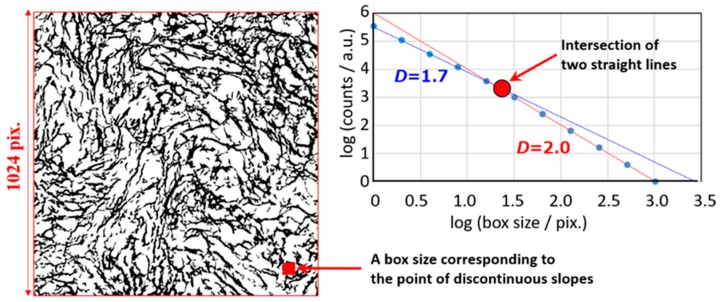
The box counting analysis for image analysis of micrographs for abnormal fibrous distribution of bone marrow. (**a**) A micrograph of bone marrow fibrosis (Reticulin and collagen fibrosis) without nucleus of hematopoietic-cells, (**b**) The box counting analysis for the micrograph shown in Figure 13a.

The box counting method was applied for a micrograph of fibrogenic hematopoietic-cells as shown in Figure 14. The micrograph was binarized for the image analysis. The results of the box counting method indicated similar values of fractal dimension with that obtained from the half full-filled box. 

However, a remarkable difference between the slopes obtained in the smaller (*D* = 1.7) and larger (*D* = 2.0) box size region was shown. In the larger box size region, any boxes include the black area, and the fractal dimension, *D* = 2, was obtained from the property of logarithmic analysis as discussed in Figure 13. On the other hand, in the smaller box size region, black and white boxes must be included, and the fractal dimension became smaller than 2 (*D* < 2). This tendency of the change in the slope is opposite to that obtained from the observation scale dependence for which the fractal dimension becomes larger in the smaller box size region. These excluded tendencies of the fractal dimension can be also a reason for difficulties in the applications without suitable knowledge. However, understanding these properties and careful approaches make it possible to have a more exact analysis.

A box size corresponding to the point of a discontinuous slope was indicated in Figure 14a. The box size clearly means the typical area of white regions, and the size must be treated as one of characterizations of the micrograph. 

**Figure 15:**
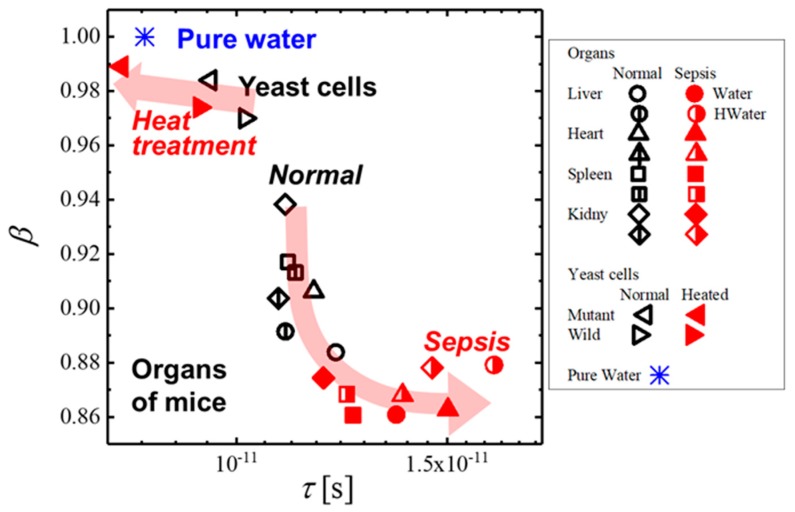
The *τ* – *β* diagram is for suspension of wild and mutant yeast cells and organs of mice with and without septic disease. Black and red symbols are used for yeast cells before and after heat treatment, respectively. Black and red symbols are also used for organs obtained from normal mice and those with septic disease, respectively. Two groups of mice are prepared to take usual mineral water or molecular hydrogen water before measurements. Symbols are listed in the explanatory notes.

Considering that the fractal dimension is just one of the three parameters determining the trajectory in the *τ* – *β* diagram, more applicable analyses using the diagram is an approach with relative changes in the plot areas with some suitable difference in water structures. Typical examples were shown in Figure 15 for yeast cell suspensions before and after heat treatment [13] and organs of clone mice [14] with and without septic disease. 

Significant differences in relaxation parameters between wild type yeast with vacuole and vacuole-deficient mutant cells was reported for MHz frequency processes, reflecting interfacial polarization, and relaxation parameters obtained for GHz frequency processes for the two cell suspensions were not very large [13]. However, some relative changes were shown in the *τ* – *β* diagram as shown in Figure 15. Plots for mutant yeast cells are shown in the upper left region because of the larger water amount and more dispersion property than those for wild type cells. Furthermore, the plot area was shifted to the upper left area with the heat treatment. It was reported that the heat treatment damaged the cell membranes [51]. This result suggests that mixtures of the intercellular buffer solution and leakage of cytoplasm bring about similar behaviors with bulk water with homogeneous water structures. More detailed explanations of relationships of cell structures and dielectric behaviors should be reported elsewhere with results of various cell systems. 

The difference between plots for two groups of mice with usual and molecular hydrogen water was not significant and is not treated in the present work. The plots for various organs were longitudinally dispersed in an area for normal organs. Water structures are dependent on organs with characteristic structures of respective tissues, and cells are characterized by those plots, especially, heterogeneities of water structures in organs. On the other hand, plots for septic mice were shifted with a direction to larger relaxation time and lower distribution parameter, and transversely dispersed in the area. Then what about “We confirmed that the macroscopic water content of each organ was not changed by the disease. Then the change in location of the plot area means changes in water structures before and after the septic disease. These results may suggest a probability of a new diagnostic evaluation of the disease. A detailed analysis of experimental data with pathological explanations should be described in future reports.

These typical usages of trajectories and plots in the *τ* – *β* diagram depend on some systematic changes of water structures. This suggests how HBN structures change with the aggregation and dispersion state, and are the effective tool to characterize water structures in complex aqueous systems, including biomaterials.

## 4. Conclusions

The application of the fractal analysis was developed in the 1980s as the universal approach applicable in extensive areas of whole natural, medical and applied sciences, and engineering. However, applications of the fractal dimension have not always been successful, since the sensitivity and accuracy have not seemed sufficient. The present work treats recent approaches of the fractal analysis with the simple model for the GHz relaxation process of dielectric spectroscopy for water structures in various aqueous materials. The effective uses of flat-ended coaxial cells and explanations with the molecular mechanism of the HBN for water structures, and the difficulties and ambiguities in conventional fractal concepts were well explained by actual data analysis of dielectric spectroscopy and box counting methods in the present work. Various usages of the *τ* – *β* diagram described here suggest that the fractal analysis of dielectric spectroscopy is certainly applicable in characterization of aqueous materials, including biological systems treated in extensive areas of science and engineering.

## Figures and Tables

**Table 1:** Fractal dimension for various aqueous systems.

Various Aqueous Materials	Fractal Dimension, *D*
Proteins	0.10 ± 92.4
Ovalbumin	0.16 ± 1.49
glass-egg	1.05 ± 0.40
Gelatin	1.77 ± 0.16
Collagen	1.74 ± 0.04
HPMC	1.57 ± 0.04
Glucose	0.78 ± 0.31
PAAm	0.13 ± 0.40
PAA	0.05 ± 0.70
PEI	1.33 ± 0.02
PAlA	1.34 ± 0.00
PVA	1.44 ± 0.15
PVP	0.90 ± 0.12
PVME	1.19 ± 0.19
PEG	1.44 ± 0.06
